# Effect of preoperative midazolam anxiolysis on anesthetic gas consumption: A randomized controlled trial

**DOI:** 10.1097/MD.0000000000049474

**Published:** 2026-06-26

**Authors:** Ersel Gulec, Mediha Turktan, Zehra Hatipoglu, Neslihan Eraslan, Dilek Ozcengiz

**Affiliations:** a Department of Anesthesiology and Reanimation, Faculty of Medicine, Cukurova University, Adana, Turkiye.

**Keywords:** anxiolytics, midazolam, preoperative period, sevoflurane, state anxiety, trait anxiety

## Abstract

**Background:**

Adult patients often experience preoperative anxiety that can influence their anesthetic requirements. We investigated the effect of a preoperative midazolam anxiolytic dose on intraoperative sevoflurane consumption.

**Methods:**

This prospective, randomized, double-blind, placebo-controlled study involved 80 patients undergoing elective surgery. Participants were randomized to receive either intravenous midazolam (0.04 mg/kg) or saline preoperatively. Preoperative anxiety assessment was conducted using the short version of the State-Trait Anxiety Inventory. The primary outcome was cumulative sevoflurane consumption within the first hour of surgery. Secondary outcomes included minimum alveolar concentration (MAC), MAC-hour, inspiratory and end-tidal sevoflurane concentrations, and hemodynamic variables.

**Results:**

Sevoflurane consumption in 60 minutes was comparable between the midazolam (mean ± standard deviation: 24.2 ± 3.9 mL) and control (25.2 ± 4.1 mL) groups (*P* = .291). The study found no statistically significant difference in mean MAC-hour values between the groups administered midazolam (3.3 ± 0.7) and saline (3.4 ± 0.7; *P* = .612). Anxiety levels and sevoflurane consumption or MAC-hour values were not significantly correlated. Hemodynamic parameters remained stable and comparable between groups.

**Conclusion:**

Preoperative anxiolytic dose of midazolam administration did not significantly reduce intraoperative sevoflurane consumption, and preoperative anxiety levels were not associated with sevoflurane requirements.

## 1. Introduction

Preoperative anxiety is a prevalent and complex psychological state that affects a substantial number of patients preparing for surgical procedures. This complex emotional state can have far-reaching consequences on both the physiological and psychological aspects of patient well-being. Physiologically, heightened anxiety levels can trigger a cascade of responses, including elevated heart rate, increased blood pressure, and alterations in neuroendocrine function. These changes may complicate anesthesia administration and raise the risk of perioperative complications.^[1–5]^ Moreover, the impact of preoperative anxiety extends well beyond the operating room. This factor can significantly influence postoperative outcomes and affect behavioral recovery, pain perception, and overall healing processes. Anxious patients often report higher postoperative pain levels and require more analgesic medication. Furthermore, preoperative anxiety has been associated with prolonged hospital stay and reduced patient satisfaction.^[6]^

Midazolam, a short-acting benzodiazepine, primarily exerts its effects via interacting with GABA_𝐴_ receptors, resulting in sedation, anxiolysis, and muscular relaxation.^[7]^ Its administration reduces the physiological stress response associated with anxiety, potentially stabilizing hemodynamic parameters and reducing the required dose of anesthetic.^[8,9]^ Despite the widespread use of midazolam for preoperative anxiolysis, data on its effects on intraoperative outcomes, particularly anesthetic gas consumption, are limited. Anxious patients may require higher anesthetic doses to achieve and maintain adequate anesthesia.^[1,10]^ Increasing the dose of midazolam at the induction of anesthesia can reduce the requirement for intraoperative anesthetic gases.^[11]^

The potential impact of preoperatively administering an anxiolytic dose of midazolam on the consumption of anesthetic gases during surgery has not been studied. This study aimed to examine how preoperative midazolam administration affects intraoperative sevoflurane consumption by reducing patient anxiety.

## 2. Methods

This prospective, randomized, double-blind, placebo-controlled trial was registered at clinicaltrials.gov (identifier, NCT05371600) on May 12, 2022. Patient recruitment was initiated after approval by the Cukurova University Clinical Research Ethics Committee (Approval No. 188/7 dated August 31, 2023). The protocol was designed and conducted in accordance with Good Clinical Practice and the Declaration of Helsinki. Before study enrollment, written informed consent was obtained from all patients. The study involved 80 patients aged 18 to 60 years with an American Society of Anesthesiologists Physical Status classification of 1 or 2. The exclusion criteria were pregnancy, a history of psychiatric disorders, use of psychotropic drugs, neurological diseases, cancer, chronic pain, and cardiovascular, respiratory, renal, hematological, and hepatic diseases. Additionally excluded were patients with uncontrolled hypertension and diabetes mellitus, as well as individuals scoring 3 or less on the Observer’s Assessment of Alertness/Sedation (OAA/S) score of ≤3.

After a 6-hour fasting period before surgery, patients were transferred to the preoperative preparation unit. Patients were allowed to hydrate before surgery by consuming clear liquids up to 2 hours before the procedure, followed by intravenous (IV) saline. All patients were monitored for peripheral oxygen saturation and noninvasive blood pressure 30 minutes before surgery. Preoperative anxiety levels were assessed using the State-Trait Anxiety Inventory (STAI) short form. The short version of the STAI comprises 10 self-report items, with 5 items focusing on state anxiety and the remaining 5 items assessing trait anxiety. The State Anxiety Inventory (STAI-S) was used to assess participants’ current anxiety levels, while the Trait Anxiety Inventory (STAI-T) was used to evaluate their general anxiety disposition. Both scales used a 4-point Likert scale ranging from “not at all” to “very much so.”^[12]^

Participants were assigned randomly to either the midazolam or control group using a computer-generated sequence in a 1:1 ratio. Assignments were concealed in opaque sealed envelopes. Group M received IV midazolam at a dose of 0.04 mg/kg along with saline (total volume 5 mL), whereas group S received an equivalent volume of saline. Patients’ alertness was assessed using the OAA/S scale.^[13]^ Drug administration and OAA/S scale assessments were performed by different investigators; patients with a score of 3 or less on the OAA/S scale were considered sedated and excluded from the study.

Before surgery, an electrocardiogram, peripheral oxygen saturation, and noninvasive blood pressure were monitored. The depth of anesthesia was continually monitored through the measurement of the GE HealthCare Entropy Module (GE Healthcare Finland, Helsinki, Finland). Anesthesia was induced using an IV bolus of propofol at a dose of 2 mg/kg. Following the induction of anesthesia, train-of-four monitoring was conducted using an acceleromyograph (Train of Four-Watch S; Organon Ireland Ltd., Dublin, Ireland). We administered rocuronium bromide IV at a dose of 0.6 mg/kg to induce neuromuscular blockade. After tracheal intubation, 1 µg/kg IV fentanyl was administered. All subjects received mechanical ventilation with end-tidal carbon dioxide levels maintained within the range of 35 to 45 mm Hg. Targeting an entropy value between 40 and 50 throughout the surgical procedure ensured consistent anesthesia depth. Sevoflurane levels were adjusted by 0.2% in response to changes in entropy, increasing when the entropy exceeded 50 and decreasing when it dropped below 40. The total gas mixture (60% nitrous oxide and 40% oxygen) flow rate was 4 L/min. An Aisys CS_2_ anesthesia device monitor (manufactured by GE HealthCare) was used to record blood pressure, heart rate, minimum alveolar concentration (MAC) value, inspiratory and end-tidal sevoflurane concentrations (%), and sevoflurane consumption (mL) at baseline and every 15 minutes during the procedure. A 0.15 mg/kg IV dose of morphine sulfate was administered 30 minutes before the end of the surgery to control postoperative pain. At the end of the procedure, anesthesia was discontinued, and the patient was extubated by spontaneous breathing and eye-opening with verbal stimulation, with a Train of Four value of 95% and an entropy value exceeding 90. An investigator who was blinded to the study group allocations administered anesthesia and recorded all pertinent data.

The primary outcome measure was the cumulative volume of sevoflurane used (in milliliters) during the first hour of anesthesia. Minimum alveolar concentration-hour (MAC-hour) was also calculated to assess anesthetic gas consumption as a secondary outcome. MAC-hour was determined by multiplying the procedure duration in hours by the weighted average end-tidal sevoflurane concentration maintained throughout the surgical phase. We calculated the weighted mean by dividing the sum of the weighted concentrations by the total time.

The formula can be expressed as follows:


$$\begin{array}{cc} Weighted\;mean\;end\text{-}tidal\;sevoflurane\;concentration\\ & =\sum \;(C_{i}\times \;T_{i})/\;\sum T_{i} \end{array}$$

where 𝑇_𝑖_ is the end-tidal sevoflurane concentration at interval 𝑖, and 𝑇_𝑖_ is the duration of interval 𝑖.

The other secondary outcomes were inspiratory and end-tidal sevoflurane concentrations, MAC, heart rate, and blood pressure.

### 2.1. Statistical analysis

In our pilot study of 10 patients, the use of sevoflurane for 60 minutes resulted in an average consumption of 26.4 (standard deviation [SD] = 4.5) mL in the control group and 23.4 (SD = 4.68) mL in the intervention group. A sample size of 38 patients per group was determined necessary to achieve a power of 80% and a significance level of 5% (two-sided). However, to account for potential data loss or dropouts, a total of 80 participants were recruited for this study. The data were analyzed statistically using IBM SPSS Statistics 26 software (IBM SPSS Statistics for Windows, Version 26.0; IBM Corp., Armonk).

We used descriptive statistics to summarize all the data. For continuous variables, we present the mean ± SD if the data follow a normal distribution. If not, the median and interquartile range are presented. Frequencies and percentages were used for categorical data. Categorical variables were compared between groups using either the chi-square test or the Fisher exact test, as appropriate. Surgeries were categorized into 4 groups: urological; general; ear, nose, and throat; and other. Independent *t* tests were used to compare sevoflurane consumption and MAC-hours between the groups because these variables were normally distributed. For variables that did not follow a normal distribution, including STAI-T, STAI-S, duration of surgery, duration of anesthesia, BMI, weight, and height, we used the Mann–Whitney *U* test for comparison. Repeated-measures analysis of variance was employed to determine the effects of time on sevoflurane consumption and identify any interactive effects of study groups and anxiety on this temporal pattern. To examine the relationship between preoperative anxiety levels and other variables, Spearman rank correlation analysis was performed. To examine how anxiety affects sevoflurane use, patients were further categorized into subgroups according to their presurgery STAI scores^[12]^:

STAI-S subgroup: high state anxiety (score ≥ 10) and low state anxiety (score < 10).

STAI-T subgroup: high trait anxiety (score ≥ 14) and low trait anxiety (score < 14).

Comparative analyses were performed within and between these anxiety subgroups to assess the impact of anxiety levels on intraoperative sevoflurane consumption and other secondary outcomes.

## 3. Results

**Figure 1.**
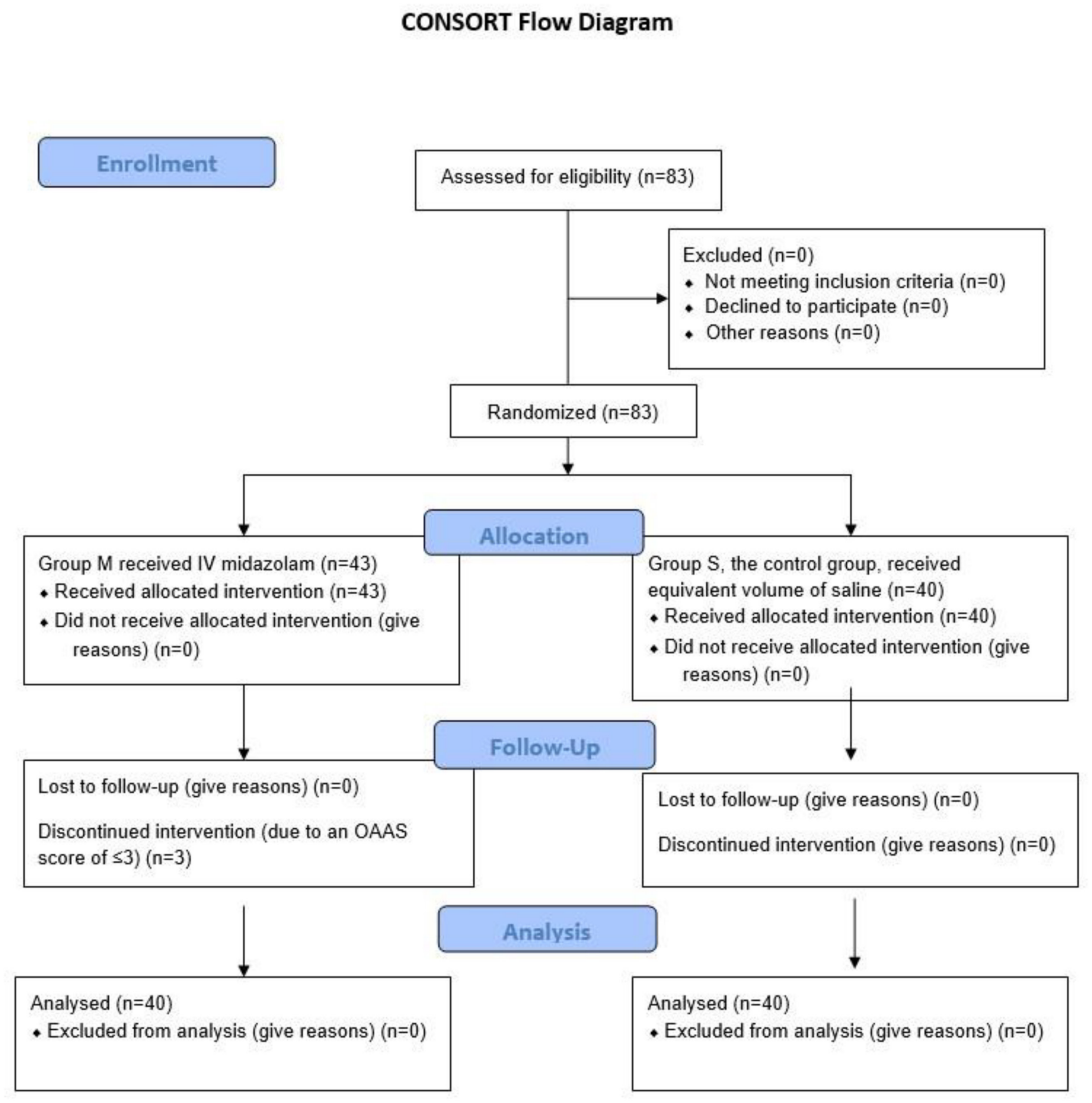
Consolidated Standards of Reporting Trials flow diagram of the study. IV = intravenous.

Among the 83 eligible patients, 3 were excluded due to an OAA/S score of ≤3. A total of 80 patients completed the study, as outlined in the Consolidated Standards of Reporting Trials flowchart (Fig. 1). Table 1 presents patient demographics and preoperative anxiety scores. STAI-T and STAI-S scores did not significantly differ between the study groups. The midazolam group scored a mean of 9.9 ± 2.6 and 8.8 ± 1.3 points in the STAI-T and STAI-S tests, whereas the saline group scored 8.9 ± 2.4 and 9.3 ± 2.0 points in both scales, respectively. The anxiety scores according to the anxiety subgroups did not significantly differ between the groups (Table 2).

**Table 1:** Demographic data about the patients.

	Group M (n = 40)	Group S (n = 40)	*P* value
Gender			
Female	20 (50.0%)	15 (37.5%)	.260
Male	20 (50.0%)	25 (62.5%)	
Age (yr)	44.0 (34.5–59.0)	50.0 (39.5–60.0)	.228
Weight (kg)	75.0 (69.5–88.0)	80.0 (74.5–88.0)	.177
Height (cm)	169 (160–179)	174 (163–181)	.170
BMI (kg/m^2^)	27.4 (25.7–28.9)	27.8 (25.5–29.0)	.912
STAI-T anxiety			
≥14	6 (15.0%)	3 (7.5%)	.288
<14	34 (85.0%)	37 (92.5%)	
STAI-S anxiety			
≥10	14 (35.0%)	19 (47.5%)	.366
<10	26 (65.0%)	21 (52.5%)	
STAI-T	10.0 (8.0–11.0)	8.0 (7.0–11.0)	.077
STAI-S	9.0 (8–10.0)	9.0 (8.0–10.5)	.246
Duration of anesthesia	146.5 (140.5–160.5)	159.0 (134.5–160.0)	.289
Duration of surgery	140.0 (131.5–153.5)	152.0 (134.5–160.0)	.464
Urological surgeries	13 (32.5%)	14 (35.0%)	.528
PCNL	4 (10.0%)	5 (12.5%)	
Nephrectomy	3 (7.5%)	2 (5.0%)	
Prostatectomy	2 (5.0%)	3 (7.5%)	
Transurethral resections	4 (10.0%)	4 (10.0%)	
General surgeries	15 (37.5%)	12 (30.0%)	
Gastric sleeve	1 (2.5%)	0 (0.0%)	
Intestinal resection	2 (5.0%)	3 (7.5%)	
Hernia repair	4 (10.0%)	3 (7.5%)	
Breast surgery	4 (10.0%)	4 (10.0%)	
Thyroid surgery	4 (10.0%)	2 (5.0%)	
ENT surgeries	9 (22.5%)	7 (17.5%)	
Rhinoplasty	5 (12.5%)	2 (5.0%)	
Tympanoplasty	2 (5.0%)	3 (7.5%)	
Mastoidectomy	2 (5.0%)	2 (5.0%)	
Other surgeries	3 (7.5%)	7 (17.5%)	

Values are presented as median (interquartile range) or frequencies and percentages.

BMI = body mass index, ENT = ear, nose, and throat, PCNL = percutaneous nephrolithiasis, STAI-S = State Anxiety Inventory, STAI-T = Trait Anxiety Inventory.

**Table 2:** Anxiety scores according to the anxiety subgroups.

	Group M (n = 40)	Group S (n = 40)	All patients (n = 80)	*P* value*
STAI-S				
≥10	10.3 ± 0.5	11.0 ± 1.3	10.7 ± 1.1	.058
<10	8.0 ± 0.9	7.8 ± 1.2	7.9 ± 1.1	.464
STAI-T				
≥14	14.2 ± 0.4	14.0 ± 0.0	14.1 ± 0.3	.516
<14	9.1 ± 2.0	8.5 ± 1.9	8.8 ± 1.2	.183

Values are presented as mean ± SD.

SD = standard deviation, STAI-S = State Anxiety Inventory, STAI-T = Trait Anxiety Inventory.

* Comparison between 2 groups.

**Figure 2.**
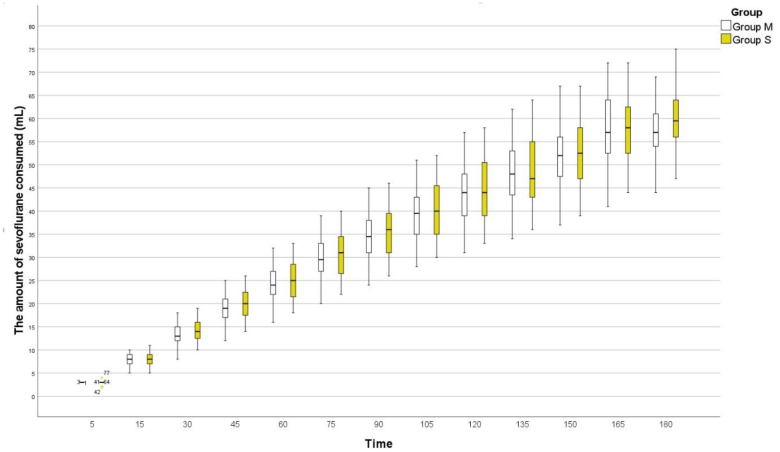
Comparison of the cumulative sevoflurane consumption between the groups at all intraoperative time points. Boxplots illustrate the horizontal line within the box (the median cumulative sevoflurane consumption), box height (25th and 75th percentiles), and whiskers extending from the edges of the box to show the range of the data, excluding outliers and potential outliers (individual points).

**Figure 3.**
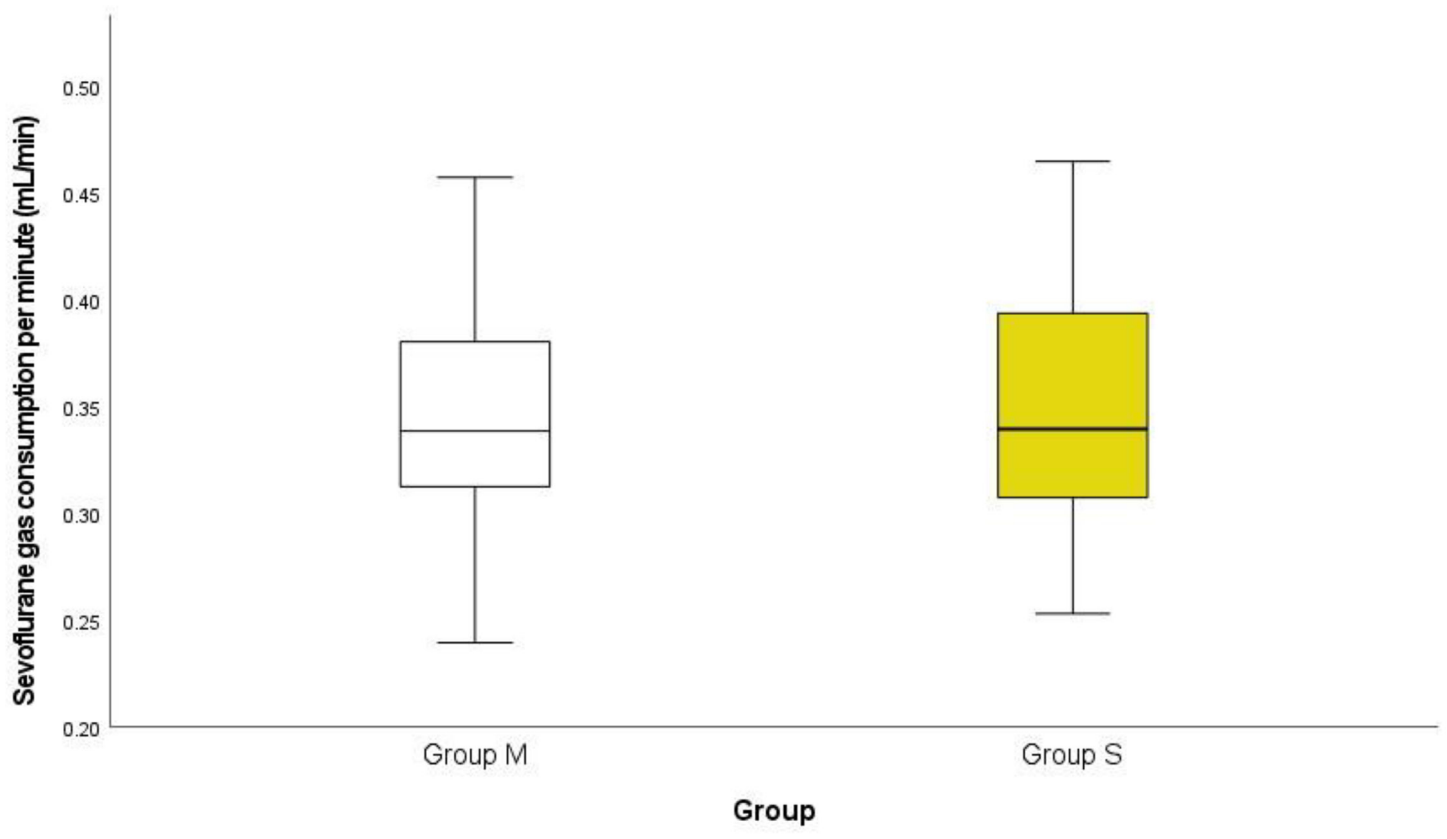
Comparison of sevoflurane consumption per minute between groups. Boxplots visualize the horizontal line within the box (the median sevoflurane consumption per minute), box height (25th and 75th percentiles), and whiskers extending from the edges of the box to show the data range, excluding outliers.

Sevoflurane use was comparable between the groups at all intraoperative time points (Fig. 2). The mean sevoflurane consumption at 60 minutes was 24.2 ± 3.9 mL in the midazolam group and 25.2 ± 4.1 mL in the control group (*P* = .291). Sevoflurane consumption per minute was similar between the groups (Fig. 3).

**Figure 4.**
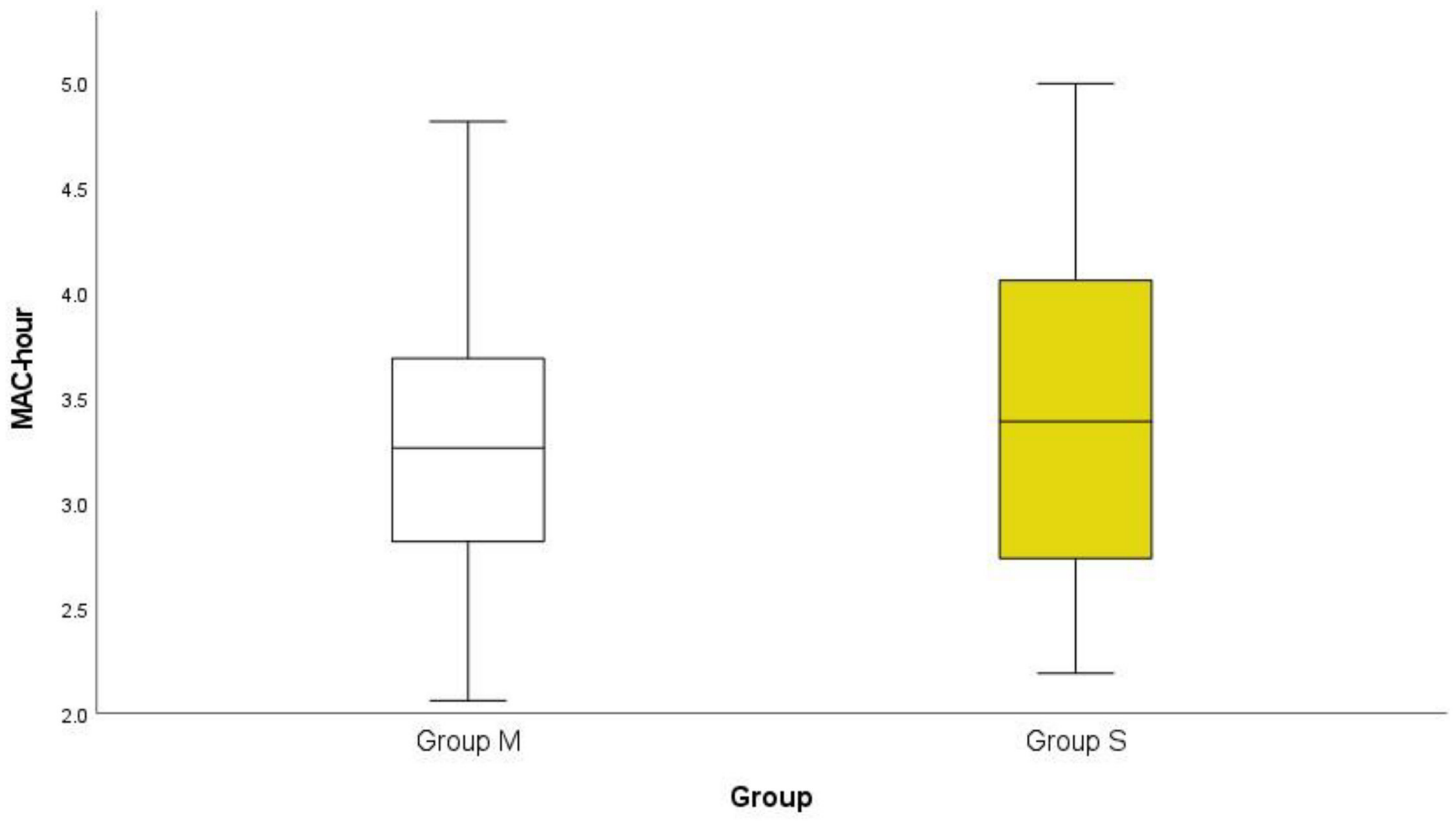
Comparison of MAC-hour values between groups. Boxplots illustrate horizontal lines within the box (the median MAC-hour values), box heights (25th and 75th percentiles), and whiskers extend from the edges of the box to show the data range, excluding outliers. MAC = minimum alveolar concentration.

The mean MAC-hour values in the midazolam and saline groups were 3.3 ± 0.7 and 3.4 ± 0.7, respectively (*P* = .612, Fig. 4). Subgroup analysis based on anxiety levels revealed no significant differences in sevoflurane consumption (volume or MAC-hour) within or between groups (Table 3).

**Table 3:** MAC-hour values according to the anxiety subgroups.

	Group M (n = 40)	Group S (n = 40)	All patients (n = 80)	*P* value*
STAI-S				
≥10	3.4 ± 0.7	3.5 ± 0.9	3.4 ± 0.8	.930
<10	3.2 ± 0.6	3.3 ± 0.6	3.3 ± 0.6	.645
*P* value†	.411	.600		
STAI-T				
≥14	3.6 ± 0.7	2.9 ± 0.5	3.4 ± 0.7	.240
<14	3.3 ± 0.7	3.4 ± 0.7	3.3 ± 0.7	.337
*P* value†	.320	.273		

Values are presented as mean ± SD.

MAC = minimum alveolar concentration, SD = standard deviation, STAI-S = State Anxiety Inventory, STAI-T = Trait Anxiety Inventory.

* Comparison between 2 groups.

† Comparison within the groups.

Neither the overall analysis nor the comparison within the study groups revealed any relationship between STAI scores or anxiety levels (by anxiety subgroups) and sevoflurane consumption (volume or MAC-hour).

Repeated measure analysis revealed a significant time effect on sevoflurane consumption (*P* = .001), but the study group interaction did not affect this (*P* = .637). In addition, the anxiety subgroups did not influence this temporal change. There were no group differences in heart rate, blood pressure, MAC value, or inspiratory or end-tidal sevoflurane concentration. In addition, no relationship was found between anxiety scores or subgroups and hemodynamic variables.

## 4. Discussion

Our findings demonstrated that preoperative midazolam administration did not reduce sevoflurane use during anesthesia. Furthermore, no significant correlation was observed between preoperative anxiety levels and intraoperative sevoflurane requirements. Several clinical implications follow from the study findings. First, although midazolam remains a reliable and effective anxiolytic, our results indicate that when anesthesia is administered using an entropy-guided technique supported by nitrous oxide, administering preoperative anxiolytic doses is unlikely to reduce intraoperative volatile anesthetic consumption. Accordingly, the decision to administer preoperative midazolam is best justified by its established benefits for patient comfort, satisfaction, and attenuation of the preoperative stress response, rather than by any anticipated savings in sevoflurane use. Clinicians who select midazolam primarily as a gas-sparing strategy are therefore unlikely to obtain a measurable benefit under these conditions.

Second, the absence of a sevoflurane-sparing effect in our cohort does not necessarily exclude such an effect in other clinical scenarios, and our results should be interpreted within the boundaries of the technique studied. The volatile-sparing effect of preoperative anxiolysis might be more evident in clinical settings that allow for greater variability in anesthetic dosing – such as low-flow anesthesia, the use of an air–oxygen carrier without nitrous oxide, or methods that do not rely on strict processed-electroencephalography titration – where the delivery protocol is less likely to obscure potential differences between study groups.

Likewise, patient populations with substantially higher baseline anxiety, or those in whom anxiety is the dominant driver of anesthetic requirement, might respond differently from the relatively low-anxiety, American Society of Anesthesiologists scoring I to II cohort examined in the present study. These considerations suggest that the clinical value of preoperative midazolam should be individualized to the anesthetic plan and the patient’s anxiety profile, rather than applied as a generalized gas-conservation measure.

Several patient factors have been identified as contributing to the need for anesthesia. Age can influence volatile uptake and MAC.^[14,15]^ Randomization balanced this factor across study arms, and subgroup analysis revealed no impact on the treatment effect. Genetic polymorphisms can influence anesthetic sensitivity, leading to variability in sevoflurane requirements, irrespective of anxiety levels.^[16]^ Baseline pain sensitivity can modulate anesthetic requirements but was not measured in this study; therefore, it may remain an unmeasured confounder to be addressed in future research.^[10]^

Anesthetic technique elements in our protocol likely shaped overall sevoflurane requirements and may have attenuated detectable between-group differences. First, administering nitrous oxide reduces the MAC of sevoflurane and lowers absolute volatile needs; this global reduction in baseline consumption leaves less dynamic range in which a premedication-related sparing effect could manifest. Second, depth-guided titration using entropy targets of 40 to 50, with protocolized adjustments in sevoflurane concentration (±0.2%), tightly constrained anesthetic delivery, minimizing interindividual variability and potentially overshadowing the subtle modulatory effects of midazolam. Third, the use of standardized induction agents (propofol 2 mg/kg and fentanyl 1 µg/kg) likely stabilized nociceptive input during the early maintenance period. In addition, the administration of morphine sulfate 30 minutes before the end of surgery to control postoperative pain may have contributed to stabilized nociception and reduced sevoflurane variability during the final intraoperative phase. Collectively, these features enhance internal control of anesthetic depth and nociception but, by narrowing the range of sevoflurane dosing, may have masked minor differences between the midazolam and control groups.

Surgical and delivery-related factors may also have limited our ability to detect a midazolam effect on sevoflurane use. Surgical category and stimulation intensity vary meaningfully across general; urologic; ear, nose, and throat; and other procedures. Although we categorized cases, the study was not stratified or powered to test surgery-type-by-treatment interactions, which could introduce heterogeneity in nociceptive input and obscure minor between-group differences. In addition, our repeated-measures analysis demonstrated a significant time effect on consumption without a group-by-time interaction, indicating that uptake and wash-in kinetics primarily governed consumption patterns rather than modulation by preoperative anxiolysis.

Previous research has established an association between preoperative anxiety and increased anesthetic requirements.^[1,10,17–25]^ Maranets and Kain^[1]^ found a correlation between higher trait anxiety and increased propofol doses, whereas Uysal et al reported an association between elevated anxiety scores, higher mean platelet volume, and increased propofol consumption in the initial stages of anesthesia.^[23]^ A study found that both state and trait anxiety were associated with increased time to achieve adequate anesthesia depth, greater propofol requirements, and more supplemental doses.^[25]^ In another study of spinal surgery patients monitored with bispectral index and neurophysiology, higher preoperative state anxiety was associated with increased intraoperative use of propofol and remifentanil.^[19]^ Findings from procedural sedation further support this trend: patients with higher preoperative anxiety required larger doses of propofol to maintain sedation.^[18,24]^ Preoperative anxiety has been shown to increase response and state entropy levels during propofol-induced unconsciousness. Higher state anxiety levels correlate with increased state entropy. These findings suggest that preoperative anxiety may influence the depth of anesthesia required during propofol induction.^[26]^ Our study failed to demonstrate a significant reduction in sevoflurane consumption following preoperative midazolam administration, suggesting that the drug’s anxiolytic properties may not directly translate to decreased anesthetic gas requirements. This discrepancy might be attributable to the distinct pharmacodynamic profiles of midazolam and sevoflurane. Although midazolam effectively reduces anxiety and stabilizes hemodynamics, its influence on anesthetic depth and gas consumption is limited. This aligns with Kil et al’s findings, which showed that preoperative anxiety affected propofol requirements but had no significant effect on sevoflurane MAC-hour.^[10]^ MAC-hour provides a potency-adjusted cumulative measure of anesthetic exposure, whereas milliliter consumption offers a direct volume-based assessment of the anesthetic agent used. The present study employed both methods to comprehensively evaluate anesthetic utilization.

An IV dose of 0.04 mg/kg administered 20 minutes before surgery has been shown to effectively alleviate anxiety.^[8]^ Sun et al demonstrated the dose-dependent anxiolytic effects of midazolam (0.02–0.06 mg/kg), with variations in response based on gender and age.^[27]^ Considering these findings, we administered a 0.04 mg/kg dose of midazolam to optimize anxiolysis while minimizing potential adverse effects.

Coadministration of midazolam during general anesthesia induction has been shown to attenuate hemodynamic responses, reduce postoperative anxiety and cortisol levels, and decrease propofol induction requirements. Given its administration during this phase, midazolam’s effects in this context should be evaluated independently of its anxiolytic properties.^[9]^ A recent study found that preoperative intramuscular midazolam, although not producing anxiolytic effects, increased the depth of anesthesia with propofol and remifentanil. This suggests midazolam may reduce anesthetic needs through different pharmacodynamic and pharmacokinetic pathways when propofol-remifentanil is used intravenously for anesthesia, rather than volatile agents.^[28]^

Melvin et al demonstrated a dose-dependent reduction in halothane requirements with increasing doses of midazolam administered during anesthesia induction.^[11]^ Notably, we administered midazolam approximately 30 minutes before induction, allowing sufficient time for its anxiolytic effects to manifest. Although this approach aimed to optimize anxiety reduction, the interval between midazolam administration and anesthesia induction might have influenced the study outcomes.

The STAI is a widely validated tool for assessing anxiety in diverse populations and surgical settings. Nevertheless, cultural variations in anxiety expression and experience may influence its accuracy. Given the STAI’s length and potential for respondent burden in a busy clinical setting, we opted to use the abbreviated STAI for anxiety assessment in this study. This decision was made to enhance patient compliance and improve data collection efficiency while maintaining adequate reliability for anxiety measurement.

This study has several limitations that should be considered when interpreting the absence of a midazolam effect. We evaluated a single midazolam dose (0.04 mg/kg) administered approximately 30 minutes before induction; consequently, our null finding cannot be attributed with certainty to an inadequate dose or to suboptimal timing, and the possibility that a higher dose or a different administration interval would alter sevoflurane consumption remains untested. The combination of 60% nitrous oxide with a fixed 4 L/min fresh gas flow reduced absolute volatile requirements and thereby narrowed the dynamic range within which a sparing effect could be detected; this delivery strategy may have attenuated a genuine between-group difference, and our findings may not generalize to air–oxygen or low-flow techniques. Similarly, entropy-guided titration tightly constrained anesthetic delivery and minimized interindividual variability, which strengthens internal control of anesthetic depth but may have simultaneously masked the subtle modulatory effect that preoperative anxiolysis could exert under less-constrained dosing.

Anxiety was assessed with the abbreviated STAI, a pragmatic instrument chosen to limit respondent burden. Although adequately reliable, its reduced granularity and susceptibility to cultural and linguistic variation may have weakened our ability to detect a relationship between anxiety and anesthetic requirement. Furthermore, although surgeries were categorized, the trial was neither stratified nor powered to test surgery-type-by-treatment interactions, so residual heterogeneity in nociceptive stimulation across procedures cannot be excluded as a factor obscuring small between-group differences. Several determinants of anesthetic requirement – including genetic polymorphisms in anesthetic sensitivity and baseline pain sensitivity – were not measured and remain potential unmeasured confounders. Finally, our primary endpoint was limited to the first operative hour, a period dominated by uptake and wash-in kinetics; effects that emerge during later maintenance or at emergence, as well as outcomes beyond gas consumption, were not captured and warrant a dedicated study. The single-center design and the stringent eligibility criteria, which excluded patients with significant comorbidity, further restrict the generalizability of our findings to broader surgical populations.

## 5. Conclusion and future prospects

Unlike studies showing a relationship between preoperative anxiety and increased propofol requirements, this study examined sevoflurane and failed to find a similar relationship, providing new evidence that expands the research. In addition, administering a preoperative anxiolytic dose of midazolam does not reduce intraoperative sevoflurane consumption during entropy-guided general anesthesia with N2O supplementation. Preoperative anxiety levels (both state and trait, measured by the short STAI) are not associated with sevoflurane requirements, whether assessed by total mL used or MAC-hour. Hemodynamic variables and end-tidal/inspiratory sevoflurane concentrations remained similar regardless of midazolam anxiolysis or anxiety level.

The primary clinical implication of our study is that, while midazolam remains an effective anxiolytic, clinicians using a similar multimodal, entropy-guided anesthetic technique should not expect routine preoperative use of midazolam to yield significant savings in sevoflurane consumption. The decision to administer midazolam can be based on its established benefits for patient comfort and satisfaction, rather than an anticipated impact on volatile agent usage.

In conclusion, we found no evidence that preoperative midazolam reduces the use of sevoflurane during anesthesia. Although midazolam is an efficient anxiolytic, it does not significantly affect anesthetic gas needs. Future research should explore its impact on other perioperative outcomes and consider alternative strategies for preoperative anxiety management.

## Author contributions

**Conceptualization:** Ersel Gulec, Zehra Hatipoglu, Neslihan Eraslan, Dilek Ozcengiz.

**Investigation:** Ersel Gulec, Mediha Turktan, Zehra Hatipoglu, Neslihan Eraslan, Dilek Ozcengiz.

**Methodology:** Ersel Gulec, Mediha Turktan, Zehra Hatipoglu, Neslihan Eraslan, Dilek Ozcengiz.

**Project administration:** Ersel Gulec.

**Data curation:** Mediha Turktan, Zehra Hatipoglu, Neslihan Eraslan.

**Resources:** Neslihan Eraslan.

**Supervision:** Dilek Ozcengiz.

**Writing – original draft:** Ersel Gulec.

**Writing – review & editing:** Ersel Gulec, Mediha Turktan, Zehra Hatipoglu, Neslihan Eraslan, Dilek Ozcengiz.
